# High Somatic Mutation and Neoantigen Burden Do Not Correlate with Decreased Progression-Free Survival in HCC Patients not Undergoing Immunotherapy

**DOI:** 10.3390/cancers11121824

**Published:** 2019-11-20

**Authors:** Angela Mauriello, Roberta Zeuli, Beatrice Cavalluzzo, Annacarmen Petrizzo, Maria Lina Tornesello, Franco M. Buonaguro, Michele Ceccarelli, Maria Tagliamonte, Luigi Buonaguro

**Affiliations:** 1 Laboratory of Cancer Immunoregulation, Istituto Nazionale per lo Studio e la Cura dei Tumori IRCCS, “Fondazione Pascale”, 80131 Naples, Italy; a.mauriello@istitutotumori.na.it (A.M.); beatrice.cavalluzzo@istitutotumori.na.it (B.C.); a.petrizzo@istitutotumori.na.it (A.P.); 2 Science and Technology Dept, University del Sannio, 82100 Benevento, Italy; robertazeuli@gmail.com (R.Z.); ceccarelli@unisannio.it (M.C.); 3 BIOGEM S.c.a.r.l., 83031 Ariano Iprino, Italy; 4 Laboratory of Molecular Biology and Viral Oncology, Istituto Nazionale per lo Studio e la Cura dei Tumori, “Fondazione Pascale”-IRCCS, 80131 Naples, Italy; m.tornesello@istitutotumori.na.it (M.L.T.); f.buonaguro@istitutotumori.na.it (F.M.B.)

**Keywords:** liver cancer, immunotherapy, cancer vaccine, personalized treatment, neoantigens

## Abstract

Cancer genome instability leads to accumulation of mutations which may result into tumor-specific mutated “neoantigens”, not be affected by central T-cell tolerance. Such neoantigens are considered the optimal target for the patient’s anti-tumor T cell immunity as well as for personalized cancer immunotherapy strategies. However, only a minor fraction of predicted neoantigens are relevant to the clinical outcome. In the present study, a prediction algorithm was applied using datasets of RNA sequencing from all 377 Hepatocellular carcinoma (HCC) patients available at The Cancer Genome Atlas (TCGA), to predict neoantigens to be presented by each patient’s autologous HLA molecules. Correlation with patients’ survival was performed on the 115 samples for whom the exact date of death was known. A total of 30 samples were used for the training set, and 85 samples were used for the validation sets. Neither the somatic mutations nor the number nor the quality of the predicted neoantigens correlate as single parameter with survival of HCC patients who do not undergo immunotherapy treatment. Furthermore, the preferential presentation of such neoantigens in the context of one of the major histocompatibility complex MHC class I molecules does not have an impact on the survival. On the contrary, the expression of Granzyme A (*GZMA*) is significantly correlated with survival and, in the context of high *GZMA*, a direct correlation between number and quality of neoantigens with survival is observed. This is in striking contrast to results described in cancer patients undergoing immunotherapy, in which a strong correlation between Tumor Mutational Burden (TMB), number of predicted neoantigens and survival has been reported.

## 1. Introduction

Tumor-specific mutations may give raise to amino acid substitutions resulting in mutated “neoantigens”. They can be recognized by tumor-specific cytotoxic T lymphocytes (CTLs) which have not subject to central tolerance induction in the thymus [1,2,3,4,5]. Therefore, mutated neoantigens are likely highly immunogenic and should be considered the optimal target for the patient’s endogenous anti-tumor T cell immunity as well as for personalized cancer immunotherapy strategies [6,7]. Indeed, tumor infiltrating lymphocytes (TILs) not only have been shown to be mostly directed towards neoantigens, but their frequency correlates to the tumor mutational burden and patients’ survival across a range of tumor types [3,8,9,10,11,12]. However, contradictory results have been recently reported about correlation of the neoantigen load with the patients’ survival in other tumors [13,14,15,16]. Moreover, the number of predicted neoantigens correlates with clinical outcomes in melanoma and lung cancer patients undergoing anti-CTLA-4 (Cytotoxic T-Lymphocyte Antigen 4) or anti-PD-1 (Programmed cell death protein 1) immune checkpoint blockade antibody therapy [17,18,19]. These findings suggest that that endogenous anti-neoantigens T cells are already present in the tumor, and their efficacy is increased by blocking inhibitory mechanisms. The relevance of the mutated neoantigens as optimal target for anti-tumor T cell response is further demonstrated by recent studies on selection of neoantigen-reactive T cells for adoptive cell therapy [20,21,22]. Finally, therapeutic cancer vaccines based on mutated neoantigens are currently evaluated in clinical trials showing significant prolonged patients’ survival [23,24,25,26].

However, true predicted neoantigens (TPNAs) are only those which do not show homology to any self-antigen, otherwise they are undetectable by the immune system [27,28,29]. Furthermore, TPNAs may show homology to pathogen-derived antigens and are a more efficient target for a pre-existing T cell memory. In support of this, the latter type of neoantigens have been identified in cancer patients either long-term survivors [30] or responders to checkpoint inhibitors [18,27].

Hepatocellular carcinoma (HCC) ranks as a medium-variable tumor, with an average mutational burden of 5 somatic mutations per Mb, corresponding to approximately 60 non-synonymous substitutions within expressed genes [31]. In such a setting of low mutational burden, mutated human leukocyte antigen (HLA) ligands appear to be rarely presented in HCC, and identification of naturally presented neo-antigens by high-efficiency mass spectrometry has failed [32].

We have recently described an improved algorithm for the prediction and validation of mutated neoantigens in HCC. Our findings show that the number of TPNAs is very limited in each patient, and only the quality of TPNAs, absence of neoantigens with homology to unrelated self-antigens and identification of neoantigens with homology to pathogen-derived peptides, correlates with long-term survival [33].

In the present study, we expanded the observation applying a similar algorithm on datasets of whole exome and RNA sequencing from all 377 HCC patients available at The Cancer Genome Atlas (TCGA). TCGA is a public data-driven platform that allows cancer researchers and bioinformaticians to search and download cancer data for genomic analyses. Neoepitopes were predicted to be presented by each patient’s autologous HLA molecules. The number of neoantigens was highly variable between different HCC patients. Survival of patients did not show any significant correlation with the number of neoantigens even with optimal characteristics, in particular, those with an affinity to the major histocompatibility complex MHC > 10 compared to the corresponding wild type epitope (differential agretopicity index, DAI > 10) and an affinity < 50 nM (high affinity) to MHC.

Interestingly, a direct correlation between the number of neoantigens and survival was observed only in patients with tumor characterized by high expression of cytotoxicity-related genes. The presence of a single predicted neoantigen with homology with epitopes of human or pathogen origin, in a tumor with multiple neoantigens, did not have any impact on survival.

Therefore, high mutation and neoantigen load do not impact survival of HCC patients who do not undergo immunotherapy.

## 2. Results

### 2.1. Tumor Mutational Burden and Survival

**Figure 1:**
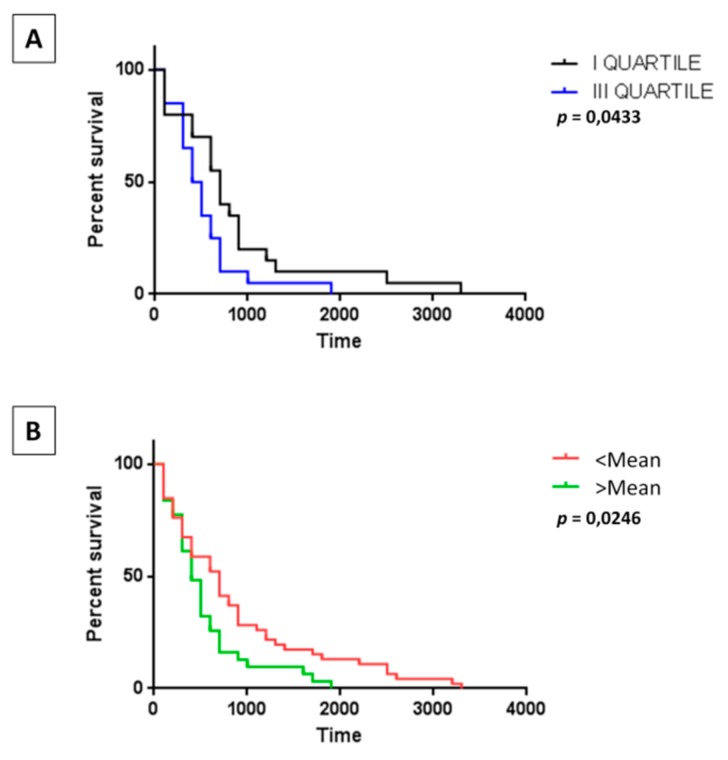
The tumor mutational burden (TMB) on survival. Survival of Hepatocellular carcinoma (HCC) patients in the I and III quartile of TMB (**A**), as well as in the patients with a TMB below and above the mean value (**B**).

The tumor mutational burden (TMB) in the HCC patients present in the TCGA databank (LIHC nr = 377) was available for 179 samples, ranging from 0.42 to 30.72 mutations per Mb (average 2.56). In particular, it was available for 76 HCC samples with a known date of death, ranging from 0.62 to 8 mutations per Mb (average 2.52). Samples were selected from the I and III quartile to correlate TMB with survival. Unexpectedly, survival curves showed an inverse correlation with a statistically significant longer survival in patients with lower TMB. This observation was confirmed also generating the survival curves with all samples and using as threshold the mean value (Figure 1A,B).

### 2.2. Neoantigen Prediction

All non-synonymous single nucleotide variations (snSNVs) identified by exome sequencing in the HCC samples with a known date of death (*n* = 115) were used to predict mutated neoantigens presented by each patient’s autologous MHC class I HLA molecules. The characteristics of the 115 samples included in the analysis are reported in Table 1. Only snSNVs identified in genes with expression levels >2 were taken into account for the prediction analysis. Peptides predicted to bind to each patient’s HLA molecules with high affinity (half maximal inhibitory concentration, IC_50_ < 500 nmol/L) were considered to be likely neoantigens. We focused only on 9-mer peptides because they account for 90% of the neoantigens that have been shown to induce T-cell responses in humans [34].

**Figure 2:**
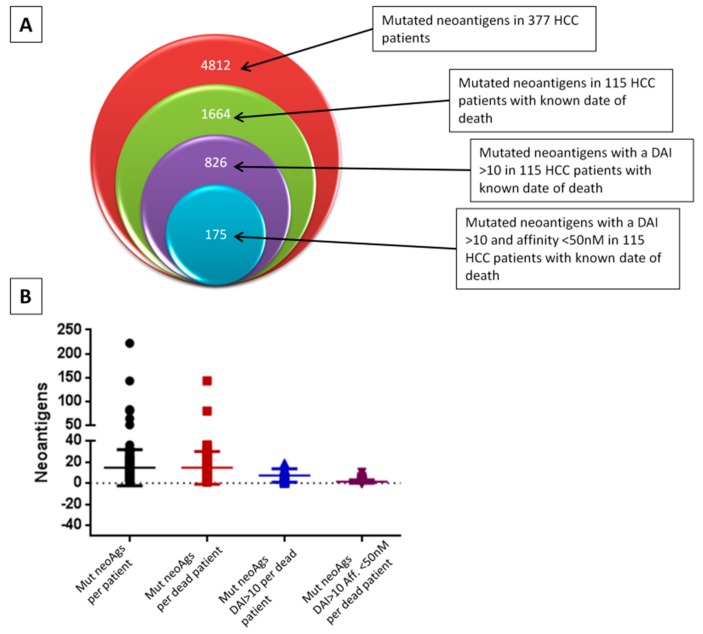
Prediction in HCC samples. (**A**) Neoantigens were predicted by NetMHCstabpan algorithm from the nsSNVs identified in the HCC samples at The Cancer Genome Atlas (TCGA) database. (**B**) Graphic representation of the number of neoantigens predicted in each HCC patient. DAI: differential agretopicity index. nsSNVs: non-synonymous single nucleotide variations.

More than sixteen-hundred mutated neoantigens (1664) have been predicted overall in the 115 HCC samples, with an average of 14.5 predicted neoantigens per samples (min 1–max 143). About half of them (nr. 826, 49.63%) showed a differential agretopicity index (DAI) >10 compared to the corresponding wild type epitope. The average of neoantigens per samples with such parameters was 7.18 (min 0–max 47). Within the latter subclass of neoantigens, 21.1% of them (nr 175) showed an extremely high affinity for the HLA <50 nM, predicting the highest prediction of antigenicity and immunogenicity. The average of neoantigens per samples with such parameters was 1.5 (min 0–max 10) and only 8 samples showed >50% of neoantigens with such highest prediction, considering the number of neoantigens with a DAI>10 (Figure 2A,B).

### 2.3. Correlation between Neoantigens and Survival

In order to assess the relationship between the number of predicted neoantigens and patient survival, the HCC samples with the lowest (I quartile) and the highest (III quartile) number of neoantigens were selected. The training set included 30 randomly selected samples, while the validation set included 85 samples.

The survival curves showed that the number of neoantigens have an inverse correlation with survival. Indeed, survival was prolonged in patients in the I quartile (*p* = 0.033), and such effect was evident also in patients below the mean value although it did not reach a statistical significance (Figure S1). The results in the training set were not different when we correlated the survival with the neoantigens with high quality, namely those with a DAI >10 and those with DAI > 10 and affinity to HLA < 50 nM. Moreover, for this analysis the survival was prolonged in HCC samples of the I quartile or below the mean value of number of neoantigens, although it did not reach a statistical significance. However, in the correlation analysis with neoantigens with DAI > 10 and affinity to HLA < 50 nM the 50% survival was significantly improved in the samples with higher number (e.g., III quartile and above the mean value), but the last observed death was significantly much later in the samples with lower number (e.g., I quartile and below the mean value) (Figure S1).

**Figure 3:**
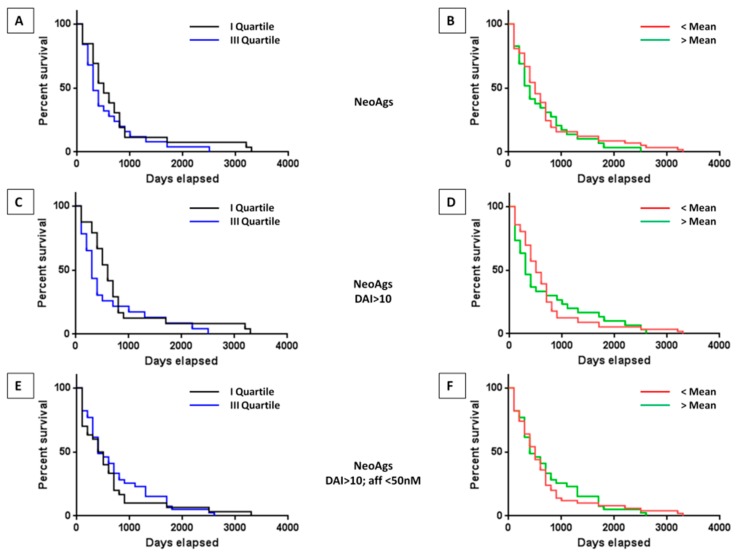
Survival of HCC patients with high and low number or quality of predicted neoantigens in the validation set, considering the value in the I and the III quartiles (**A**,**C**,**E**) as well as the value below and above the mean (**B**,**D**,**F**). Neoantigens: overall predicted neoantigens (**A**,**B**). Neoantigens DAI > 10: predicted neoantigens with a differential agretopicity index (DAI) > 10 compared to the corresponding wild type epitope (**C**,**D**). Neoantigens DAI > 10; affinity < 50 nM: predicted neoantigens as before, with a value of affinity < 50 nM (**E**,**F**).

**Figure 4:**
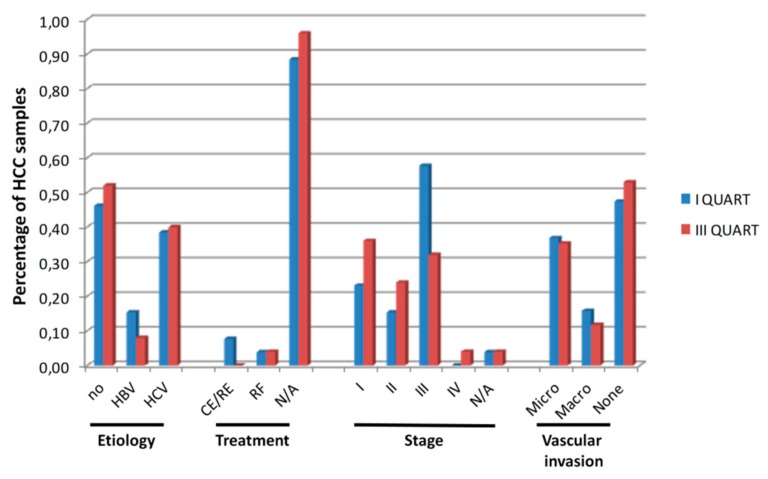
Clinical characteristics of HCC samples. The percentage of HCC samples with the indicated characteristics in the I and III quartiles of number of neoantigens is shown. CE/RE = chemo and radioembolization; RF = radiofrequency. HBV:Hepatitis B virus; HCV: Hepatitis C virus; NA: Not available no:Not viral

The same analysis performed in the validation set confirmed the data obtained in the training set, without reaching a statistical significance (Figure 3A–F). Indeed, regardless the quality of the neoantigens used in the comparison analysis, the overall survival in the samples with lower number of neoantigens (e.g., I quartile and below the mean value) was longer compare to samples with higher number of antigens, although the difference between the two groups did not reach a statistical significance (Figure 3A–F). Such a correlation was not biased by clinical parameters (e.g., viral etiology, treatment, stage and vascular invasion) which did not show a significant difference between the samples falling into the I and III quartiles (Figure 4).

### 2.4. Impact of HLA on the Survival

**Figure 5:**
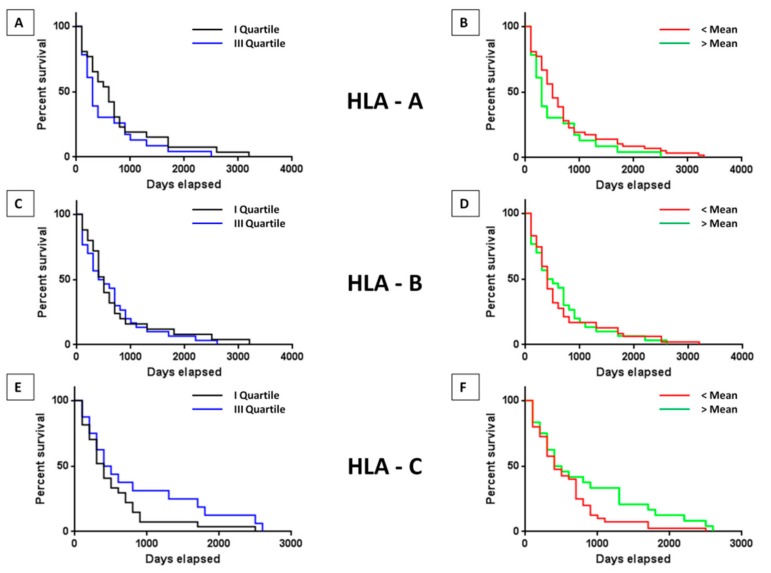
HLA-associated neoantigens and survival. Survival of HCC patients with high and low number or quality of predicted neoantigens associated with the three main HLA alleles in the validation set, considering the value in the I and the III quartiles (**A**,**C**,**E**) as well as the value below and above the mean (**B**,**D**,**F**).

In order to evaluate whether the survival is affected by the alleles presenting the predicted neoantigens, the number of predicted neoantigens associated with the HLA A, B or C alleles in HCC samples was correlated with survival. The training set showed a statistically significant increased survival only in the samples characterized by a number of neoantigens associated with HLA-C higher than the mean value (*p* = 0.02) (Figure S2). Such result was confirmed in the validation set, although it did not reach the statistical significance (Figure 4). On the contrary, no difference in survival was observed in the samples, regardless of the number of neoantigens associated with HLA-A or HLA-B, neither in the training nor in the validation set (Figure S2 and Figure 5A–F).

### 2.5. Impact of Tumor Microenvironment of Survival

In order to assess the correlation between the number of predicted neoantigens and tumor infiltrating immune cells in each HCC patient, heat maps were generated using expression level values of genes characterizing effector (CD4^+^ and CD8^+^ T cells) and regulatory (Tregs and MDSC) cells (Figure S3A–D). In each heat map two clusters of HCC samples were selected according to the boostrap analysis, identifying samples with high and low gene expression values. Subsequently, the number of predicted neoantigens in each of the samples of the two clusters were plotted. The results showed a similar number of neoantigens in the samples falling in the two clusters of low and high gene expression values for all four immune cells, suggesting that there is no correlation between quantity or quality of neoantigens and tumor immune infiltration (Figure S4). Such an observation was further confirmed by the lack of correlation between expression levels of effector or regulatory cells and survival (Figure S5A–D).

We therefore took into consideration the individual expression levels of the main mediator of the cytotoxic activity of CD8^+^ T cells (e.g., Granzyme A-GZMA), in combination with the marker of CD4^+^ T regulatory cells (e.g., FoxP3) and with the checkpoint molecule PD-L1 (Programmed cell death protein ligand 1).

**Figure 6:**
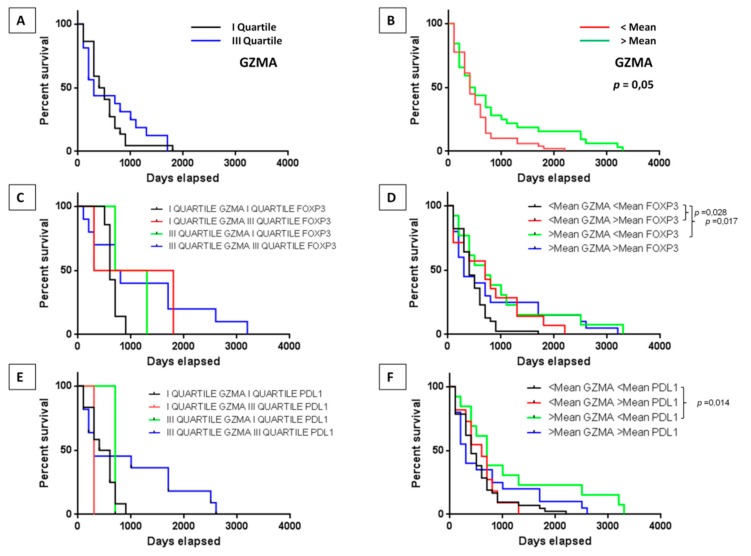
Microenvironment and survival. Survival of HCC patients with high and low gene expression of Granzyme alone or in association with FoxP3 or PDL1 in the validation set. The association was evaluated considering the value in the I and the III quartiles (**A**,**C**,**E**) as well as the value below and above the mean (**B**,**D**,**F**).

Survival curves in the samples with low and high expression of *GZMA* did not show statistically significant differences in the training set, although a trend of improved survival in the samples with high expression level was observed (Figure S6). On the contrary, the statistical difference was reached in the validation set, showing an improved survival in samples with expression levels of *GZMA* higher than the mean value (*p* = 0.05) (Figure 6A,B). The increased survival in the latter samples was further confirmed in the subset of samples with low expression of *FoxP3* or of *PD-L1* (*p* = 0.017 and *p* = 0.014, respectively) (Figure 6C–F).

### 2.6. Correlation of Neoantigens and Tumor Microenvironment

**Figure 7:**
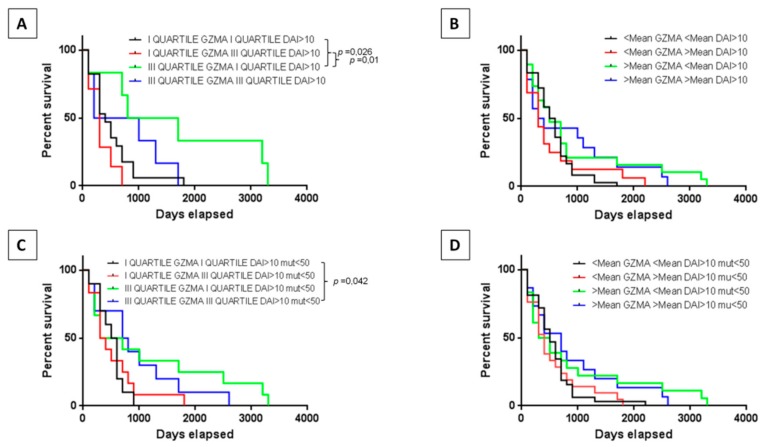
Neoantigens and survival. Survival of HCC patients correlating the *GZMA* gene expression with high and low number of neoantigens DAI >10 or neoantigens DAI >10 and affinity <50 nM in the validation set. The association was evaluated considering the value in the I and the III quartiles (**A**,**C**) as well as the value below and above the mean (**B**,**D**).

The next step was to assess the role of the neoantigens in the survival of patients according to the tumor infiltration by immune cells. The training set did not show any statistically significant change in the survival in patients regardless of the expression levels of *GZMA* in combination with number of high-quality neoantigens (e.g., DAI > 10 or DAI > 10 and affinity to HLA < 50 nM). However, a trend in the increased survival was observed in all patients characterized by high expression of *GZMA* (Figure S7). On the contrary, results reached the statistical significance in the validation set. In particular, patients in the III quartile of *GZMA* expression showed a significant prolonged survival compared to those in the I quartile considering low or high levels of neoantigens (Figure 7A–D).

When considering the impact of the number of neoantigens in the survival of patients according to the combined expression of *GZMA* and *FoxP3* or *PDL1*, no statistical difference was observed between groups.

### 2.7. Sequence Homology Analysis with Known Epitopes

The final step was to verify whether the HCC samples showed predicted neoantigens with sequence homology to any known self or pathogen-derived antigen reported in the literature, through a blast search against epitopes in the Immune Epitope Database (IEDB; http://www.iedb.org/).

According to such analysis, 10 samples showed a predicted neoantigen with sequence homology to pathogen-derived antigens, and 17 samples showed a predicted neoantigen with sequence homology to unrelated self-antigens. Four samples showed predicted neoantigens with sequence homology to both a pathogen-derived and an unrelated self-antigen (Tables S1 and S2). The number of identical residues in the T-cell receptor (TCR) binding positions (p1, p4, p5 and p8) ranged between 2 and 4; while considering all the 9 epitope residues, the number of identical residues ranged between 5 and 7 (Figure S8; Tables S3 and S4).

**Figure 8:**
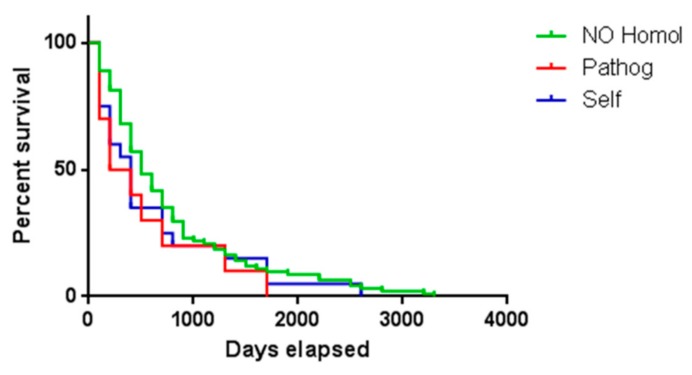
Similar to published epitopes and survival. Survival of HCC patients with neoantigens showing homology either to self or pathogen-derived epitopes from iedb.org or to any known antigen.

In order to verify whether the homology between predicted neoantigens and any known self or pathogen-derived antigen had an impact on survival, HCC samples were divided in three groups according to the presence or not of predicted neoantigens with homology to known antigens. The survival analysis included 88 samples (no homology, 76.5%), 10 samples (homology to pathogens, 8.7%), 17 samples (homology to self-antigens, 14.8%). The results showed that the presentation by the tumor of a single neoantigen, among many others, with homology to other know antigens did not have an impact on patients’ survival (Figure 8).

## 3. Discussion

In the present study datasets of all 377 HCC patients available from The Cancer Genome Atlas (TCGA) were analyzed to predict neoantigens to be presented by each patient’s autologous HLA molecules. However, in order to make accurate correlations with patients’ survival, all the evaluations were then performed only on the 115 samples for which the exact date of death is reported. Indeed, the date of follow up reported for the majority of samples in the TCGA indicates only when the patients were lost to follow up but does not give any clue about the actual survival of the patient. Therefore, the latter cannot be used for correlating the number and quality of neoantigens and patients’ survival.

Contrary to what expected and reported for other tumors, the TMB did not show an impact on the survival of HCC patients and a lower TMB correlated with a prolonged survival. Indeed, the Tumor Mutational Burden (TMB) is strictly correlated to the number of neoantigens arising in a tumor, representing a predictive marker of responsiveness to immunotherapy [35,36]. However, this is not a general rule, as shown for glioma and multiple myeloma [37].

The average number of predicted neoantigens in the HCC samples with an exact date of death was 14.5. About 50% of them showed a DAI >10 compared to the corresponding wild type epitope and, of these, about 1/5 showed an affinity for the HLA <50 nM, which indicated a very high prediction of antigen presentation, antigenicity and immunogenicity. The inter-sample variation was extremely high. The number of predicted neoantigens, regardless of their predicted quality, did not show a trend of a direct correlation with survival. Indeed, the overall survival in the samples with lower number of neoantigens (e.g., I quartile and below the mean value) was longer compared to samples with higher number of antigens, although the difference between the two groups did not reach a statistical significance. This disagrees with what was previously reported in pancreatic cancer [30], bladder cancer [15] and renal cancer [13]. The lack of information about the HCC etiology (e.g., HBV, HCV, non-viral) in the TCGA dataset does not allow a stratification of the samples nor the evaluation of its impact on both tumor mutational burden and neoantigen load.

Considering the role of the HLA alleles, the results showed no correlation between the number of predictive neoantigens associated to the three major alleles and survival, suggesting that such a parameter does not represent a predictive factor for cancer patients’ survival.

Interestingly, the number of neoantigens, irrespective of their characteristics, did not show a statistically significant difference in patients with high or low infiltration of effector or suppressive immune cells. This would suggest that the number of infiltrating cells is not driven by the number of neoantigens presented by the HCC. However, a higher gene expression of *GZMA* in the tumor infiltrating lymphocytes correlated with prolonged survival, and a lower expression of FoxP3 or PDL1 further improved the patients’ survival. This confirms that a more active state of effector CD8^+^ T cells, combined to a lower infiltration of CD4^+^ T regulatory cells or a low expression of the PDL1, provides a more effective tumor micro-environment with an improved survival.

The number of predicted neoantigens showed a direct impact on patients’ survival when combined to *GZMA* gene expression. Patients in the III quartile of *GZMA* expression showed a significant prolonged survival compared to those in the I quartile considering low or high levels of neoantigens. Such results would suggest that when the cytotoxic activity of the infiltrating lymphocytes is high, this is predictive of better prognosis irrespective of the number of neoantigens presented by the tumor. Indeed, even a low number of neoantigens would be sufficient to drive an effective anti-tumor response. On the contrary, when such cytotoxic activity is lower, the number of neoantigens does not appear to have an impact on the patients’ survival.

We further characterized the predicted neoantigens by assessing homology with any known epitopes of human or pathogen origin [33]. Indeed, neoantigens sharing homology with an epitope of human origin are immunogenically “self” antigens and should not elicit any immune response. On the contrary, neoantigens sharing homology with an epitope of pathogen origin should be able to elicit a stronger anti-tumor immune response, if the patient has a pre-existing immunity for that specific pathogen-derived epitope. To this aim, a blast search in the Immune Epitope Database (IEDB; http://www.iedb.org/) showed that 10 samples showed a predicted neoantigens with sequence homology to pathogen-derived antigens, and 17 samples showed a predicted neoantigens with sequence homology to unrelated self-antigens. In particular, four samples showed a predicted neoantigen with homology to Hepatitis C virus (HCV) epitopes, which is one of the main risk factors for HCC development, one sample showed a predicted neoantigen with homology to E6 protein of Human papillomavirus (HPV), which is almost ubiquitous in the human population. Interestingly, the sequence homology between predicted neoantigens and the epitopes covered not only the TCR binding positions (p1, p4, p5 and p8) but also the HLA binding positions. Such homology at the TCR binding positions is predictive of a cross-antigenic properties of antigens, as recently described by our group [33].

Our results showed that the identification of a single predicted neoantigens with homology to self or pathogen-derived antigens did not have any positive nor negative impact on patients’ survival. Obviously, we did not have the possibility to verify whether a memory T cell response against the pathogen-derived antigens was present in the HCC patients. However, a plausible explanation could be that in a tumor with multiple predicted neoantigens, the biological effect of a single neoantigen with homology to either to unrelated self- or to pathogen-derived antigen cannot impact on the patient’s overall survival.

In conclusion, we showed that neither the TMB nor the number nor the quality of the predicted neoantigens are associated with a prolonged survival in HCC patients not undergoing immunotherapy treatment.

This is in contrast with results in melanoma and lung cancer patients undergoing immunotherapy [17,18]. These apparently contradictory results might be due to the small number of mutations and low neoepitope load in HCC patients. However, most importantly, this might be because HCC patients in the TCGA had never received immune checkpoint inhibitors such as anti-PD-1 antibody. In fact, all the patients in the database had received standard therapy with surgery (tumor resection), loco-regional treatments (tumor ablation) or systemic therapy with MAP-Kinase inhibitors (Sorafenib), according to evidence-based clinical practice guidelines for HCC [38].

Whether high mutation/neoepitope load shows any positive association with prognosis in HCC patients who do receive immune checkpoint blockade therapy remains to be investigated.

Nevertheless, we found that HCC patients with a high potential neoepitope, coupled with a favorable tumor microenvironment, as assessed by high expression of *GZMA*, low expression of *FoxP3* and *PDL1*, tended to have a prolonged survival. Therefore, these results indicate that intact neoantigen presentation machinery, but not mutation/neoantigen load alone, may contribute to a more favorable prognosis in HCC patients.

It is confirmed that predicted neoantigens may show high homology to unrelated self or pathogen-derived antigens. However, it does not have an impact on patients’ survival if the neoantigen with such characteristics is only one of the many presented by the tumor. Indeed, our recent results showed an impact on survival only when the number of neoantigens with homology to pathogen-derived antigens was relevant over the total [33].

The lack of information in TCGA about the treatment may represent a limitation in the interpretation of the correlation results on survival described in the present study. Nevertheless, such a limitation is significantly mitigated by the information about the stage of disease available for 95% of the samples and which represents the driver for selecting the treatment applied to HCC patients. The observation that patients with low and high number of neoantigens (first and third quartile, respectively) do not segregate in different stages as well as the distribution of samples in stages I–III is equal or similar, represent a strong indication that treatment does not represent a bias in the observed survival data. Therefore, the findings of the present study represent a first clear demonstration of lack of correlation between number/quality of neoantigens and survival in HCC patients not undergoing immunotherapy.

## 4. Materials and Methods

### 4.1. Clinical Cohorts and Outcome Assessments

Cohorts of patients with hepatocellular carcinoma (LIHC *n* = 377) were identified from TCGA (https://www.cancer.gov/about-nci/organization/ccg/research/structural-genomics/tcga) and served as datasets for neoantigen prediction and evaluation of the association between predicted neoantigens and survival. Patient survival was the primary outcome measure in this study.

The study does not need ethics approval. The corresponding author has received consent for publication. Data and material are available upon request.

### 4.2. Neopeptide Prediction and DAI Analysis

Epitope prediction was performed for non-synonymous somatic SNVs using prediction tools available at http://www.cbs.dtu.dk/services/. The NetMHCpan version 4.0 (http://www.cbs.dtu.dk/services/NetMHCpan/) was used to predict MHC class I HLA-A restricted epitopes based on the combination of different parameters and ranked on prediction values (%rank). Predicted epitopes were selected based on % rank ≤2. To calculate DAI, MHC-I affinity was predicted for mutant and wild type peptide pairs arising from the same mutation and differing by a single amino acid. The DAI of each mutant peptide was calculated by subtraction of its predicted binding affinity from the value of the corresponding wild-type peptide [39].

### 4.3. Epitope Prediction and Sequence Homology Analysis

The Immune Epitope Database (IEDB; http://www.iedb.org/) was used for analysis of sequence homology to experimentally validated human and pathogen-derived antigens. Known antigens with homology >70% to mutated antigens were identified, but only those with matching aa residues at the TCR binding positions (positions 1, 4, 5 and 8) were selected for subsequent analyses. Homologous validated antigens were subsequently confirmed by the NetMHCstabpan version 1.0 (http://www.cbs.dtu.dk/services/NetMHCstabpan/).

### 4.4. Assessment of Tumor Infiltrating Immune Cells

Expression values of gene sets specific for the CD8^+^ T cells, CD4^+^ T cells, Tregs, MDSC (according to the Cancer Immunome Atlas-https://tcia.at/home) were used to generate supervised heatmaps according to a hierarchical clustering. The stability of clusters was assessed by bootstrap resampling with 100 iterations. The levels of gene expression were considered as evidence for tumor infiltration by each of the cells. All the samples in the upper clusters (above the arrow in Figure S3A–D) were considered as HIGH, and all those in the lower clusters (below the arrow in Figure S3A–D) were considered as LOW infiltrated samples. Alternatively, expression levels of individual immune related genes *GZMA*, *FoxP3* and *PDL1* were used.

### 4.5. Statistical Analysis

Comparisons between individual data points were performed with the unpaired two-sided Student’s *t*-test and ANOVA, as appropriate. Normally distributed data were represented as mean ± S.E.M. Survival analyses were generated by Kaplan–Meyer’s curves. All *p* values were two-tailed and considered significant if less than 0.05. Data analysis was performed using the statistical software Prism 7.0, GraphPad software (GraphPad Software, 2365 Northside Dr., Suite 560, San Diego, CA, USA).

## 5. Conclusions

The results show that although TMB, number and quality of neoantigens do not correlate with survival in HCC who do not undergo immunotherapy, the identification of high quality neoantigens in HCC remains the highest priority for predicting patients’ survival as well as developing highly active personalized immunotherapy (e.g., cancer vaccines as well as adoptive cell transfer).

## Figures and Tables

**Table 1:** Charateristics of Hepatocellular carcinoma (HCC) patients in The Cancer Genome Atlas (TCGA) included in the analysis of the present study.

Group	Characteristics
sex	
male	61.50%
female	38.50%
age	18–90 years (average 61.5 years)
etiology	
HBV	19.20%
HCV	36.10%
Non viral	44.60%
stage	
I	33%
II	19.20%
III	34.60%
IV	2.30%
N/A	10.80%
Treatment	
RF	4.60%
CE	11.50%
RE	3.80%
N/A	80%

RF = radiofrequency; CE = chemoembolization; RE = radioembolization. HBV: Hepatitis B virus; HCV: Hepatitis C virus; N/A: not available.

## Supplementary Materials

The following are available online at https://www.mdpi.com/2072-6694/11/12/1824/s1, Figure S1: Neoantigens and survival, Figure S2: HLA-associated neoantigens and survival, Figure S3: Heat map of gene expression for tumor infiltrating immune cells, Figure S4: Correlation between neoantigens and tumor infiltrating cells, Figure S5: Tumor infiltrating cells and survival, Figure S6: Tumor microenvironment and survival, Figure S7: *GZMA*, neoantigens and survival, Figure S8: Sequence homology between neoantigens and published epitopes, Table S1: Mutated neoantigens with homology to pathogen derived antigens, Table S2: Mutated neoantigens with homology to unrelated self-antigens, Table S3: Alignment of each predicted mutated neoantigen and the homologous pathogen derived antigen from iedb.org, Table S4: Alignment of each predicted mutated neoantigen and the homologous cellular self-antigen from iedb.org.
