# Supplementary material for: High Somatic Mutation and Neoantigen Burden Do Not Correlate with Decreased Progression-Free Survival in HCC Patients not Undergoing Immunotherapy
Source: Cancers (Basel). 2019 Nov 20;11(12):1824. doi: 10.3390/cancers11121824 (PMC6966682; doi:10.3390/cancers11121824)
Supplement: Supplementary file 1 [file cancers-11-01824-s001.pdf]

Supplementary Materials: High Somatic Mutation and Neoantigen Burden do not Correlate with Decreased Progression-Free Survival in HCC Patients not Undergoing Immunotherapy

Angela Mauriello, Roberta Zeuli, Beatrice Cavalluzzo, Annacarmen Petrizzo, Maria Lina Tornesello, Franco M. Buonaguro, Michele Ceccarelli, Maria Tagliamonte and Luigi Buonaguro

Table S1. Mutated neoantigens with homology to pathogen derived antigens.

SAMPLE	Nr neoAg DAI>10	Nr neoAg DAI>10 mut <50	Pathogen	Homologous TCR Binding	Homologous Total
TCGA-2Y-A9H1	11	2	dengue	3 aa	5 aa
TCGA-CC-A5UD	12	3	TAX HTLV II	3 aa	5 aa
TCGA-DD-A39X	5	1	malaria	3 aa	7 aa
TCGA-DD-A3A4	9	1	Protein E6	2 aa	5 aa
TCGA-DD-AACG	7	1	HCV	2 aa	5 aa
TCGA-DD-AADF	19	1	HCV	3 aa	5 aa
TCGA-DD-AADM	10	2	HCV	3 aa	5 aa
TCGA-FV-A3R2	7	1	Trip. Cru	2 aa	6 aa
TCGA-G3-A7M9	11	0	M tuber	3 aa	5 aa
TCGA-GJ-A6C0	8	1	HCV	2 aa	6 aa

Table S2. Mutated neoantigens with homology to unrelated self antigens.

SAMPLE	NeoAg DAI>10	NeoAg DAI>10 mut <50	Homologous TCR Binding	Homologous Total
TCGA-2Y-A9H1	11	2	3 aa	7 aa
TCGA-4R-AA8I	47	2	3 aa	6 aa
TCGA-BC-A10W	8	0	3 aa	7 aa
TCGA-CC-5258	8	1	3 aa	7 aa
TCGA-CC-5264	10	2	3 aa	6 aa
TCGA-CC-A5UD	12	3	3 aa	6 aa
TCGA-CC-A8HV	12	2	3 aa	6 aa
TCGA-DD-A116	9	3	3 aa	7 aa
TCGA-DD-AACC	14	2	3 aa	6 aa
TCGA-DD-AACD	12	1	2 aa	7 aa
TCGA-DD-AACL	11	3	3 aa	7 aa
TCGA-EP-A2KC	6	2	3 aa	6 aa
TCGA-G3-A7M9	11	0	3 aa	6 aa
TCGA-GJ-A6C0	8	1	3 aa	7 aa
TCGA-KR-A7K0	8	1	3 aa	6 aa
TCGA-XR-A8TF	6	1	3 aa	6 aa
TCGA-ZP-A9D4	7	1	4 aa	7 aa

Table S3. Alignment of each predicted mutated neoantigen and the homologous pathogen derived antigen from iedb.org. Green letters, indicate identical residues between the two sequences; Red letters, indicate different residues between the two sequences.

HCC SAMPLE	Peptide	p1	p2	p3	p4	p5	p6	p7	p8	p9
TCGA-DD-A39X	mut	L	L	A	V	S	S	H	W	L
	malaria	L	L	A	V	S	S	I	L	L
TCGA-FV-A3R2	mut	G	A	L	A	L	A	Q	V	L
	Trip. Cru	R	A	L	S	L	A	A	V	L
TCGA-DD-A3A4	mut	M	A	F	S	D	L	T	S	M
	Protein E6	F	A	F	S	D	L	C	I	V

TCGA-G3-A7M9*	mut	T	L	Y	D	G	P	N	A	R
	M tuber	L	L	Y	D	G	S	F	A	V
TCGA-GJ-A6C0*	mut	M	A	P	L	G	G	A	P	L
	HCV	Y	I	P	L	V	G	A	P	L
TCGA-CC-A5UD*	mut	S	E	I	G	F	G	E	S	L
	TAX HTLV II	H	F	P	G	F	G	Q	S	L
TCGA-DD-AACG	mut	S	I	I	S	F	D	P	A	V
	HCV	I	L	D	S	F	D	P	L	V
TCGA-DD-AADF	mut	R	A	S	G	K	A	Q	P	L
	HCV	P	F	Y	G	K	A	I	P	L
TCGA-DD-AADM	mut	T	A	E	E	Q	G	A	Q	L
	HCV	P	Y	I	E	Q	G	M	Q	L
TCGA-2Y-A9H1*	mut	S	S	V	P	V	S	I	P	K
	dengue	G	L	F	P	V	S	I	P	I

*samples with neoantigens with homology also to cellular self antigens.

Table S4. Alignment of each predicted mutated neoantigen and the homologous cellular self antigen from iedb.org. Green letters, indicate identical residues between the two sequences; Red letters, indicate different residues between the two sequences.

HCC SAMPLE	Peptide	p1	p2	p3	p4	p5	p6	p7	p8	p9
TCGA-2Y-A9H1*	mut	S	S	A	S	V	P	P	N	K
	self	M	S	A	S	V	H	P	N	K
TCGA-4R-AA8I	mut	G	E	L	H	A	H	T	Q	A
	self	G	E	L	E	A	E	R	Q	A
TCGA-BC-A10W	mut	R	R	T	E	V	A	H	A	L
	self	R	R	V	E	I	A	H	A	L
TCGA-CC-5258	mut	F	L	H	S	K	G	L	M	Y
	self	F	L	D	S	K	G	L	E	Y
TCGA-CC-5264	mut	F	S	S	P	T	G	N	H	V
	self	Y	S	Y	P	T	G	N	H	T
TCGA-CC-A5UD*	mut	N	E	V	I	K	L	Q	Q	L
	self	N	E	V	I	G	I	R	Q	L
TCGA-CC-A8HV	mut	S	L	L	T	L	Y	L	D	Y
	self	R	M	L	T	L	W	L	D	Y
TCGA-DD-A116	mut	S	E	V	E	L	F	R	S	F
	self	S	E	I	E	L	F	R	V	F
TCGA-DD-AACC	mut	L	A	R	E	R	R	A	V	L
	self	L	A	P	E	R	R	S	T	L
TCGA-DD-AACD	mut	A	V	L	Q	S	G	A	P	I
	self	K	V	L	D	S	G	A	P	I
TCGA-DD-AACL	mut	A	A	F	I	I	T	S	D	R
	self	A	A	F	I	I	G	S	G	R
TCGA-EP-A2KC	mut	E	L	V	D	R	A	G	R	R
	self	Q	L	V	D	R	Q	N	R	R
TCGA-G3-A7M9*	mut	Y	L	K	P	P	N	L	L	L
	self	Y	L	R	P	P	N	T	S	L
TCGA-GJ-A6C0*	mut	Q	R	F	L	F	P	P	G	I
	self	N	R	F	L	F	P	E	G	I
TCGA-KR-A7K0	mut	V	S	I	L	G	D	T	L	L
	self	V	S	I	L	G	D	E	V	F
TCGA-XR-A8TF	mut	E	E	A	A	S	S	L	K	Y
	self	R	E	A	A	S	R	L	K	W
TCGA-ZP-A9D4	mut	K	V	T	S	S	D	T	S	L
	self	K	V	I	S	S	N	T	S	L

*samples with neoantigens with homology also to cellular pathogen-derived antigens.

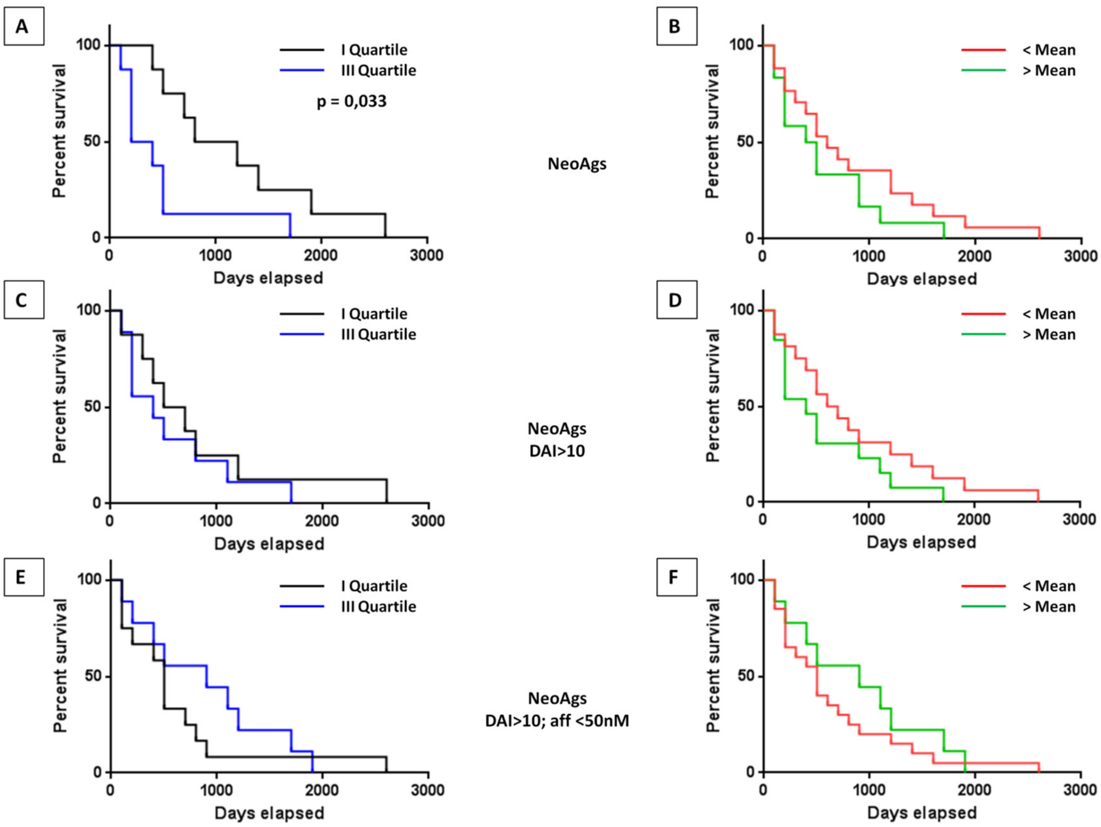

Figure S1. Neoantigens and survival.

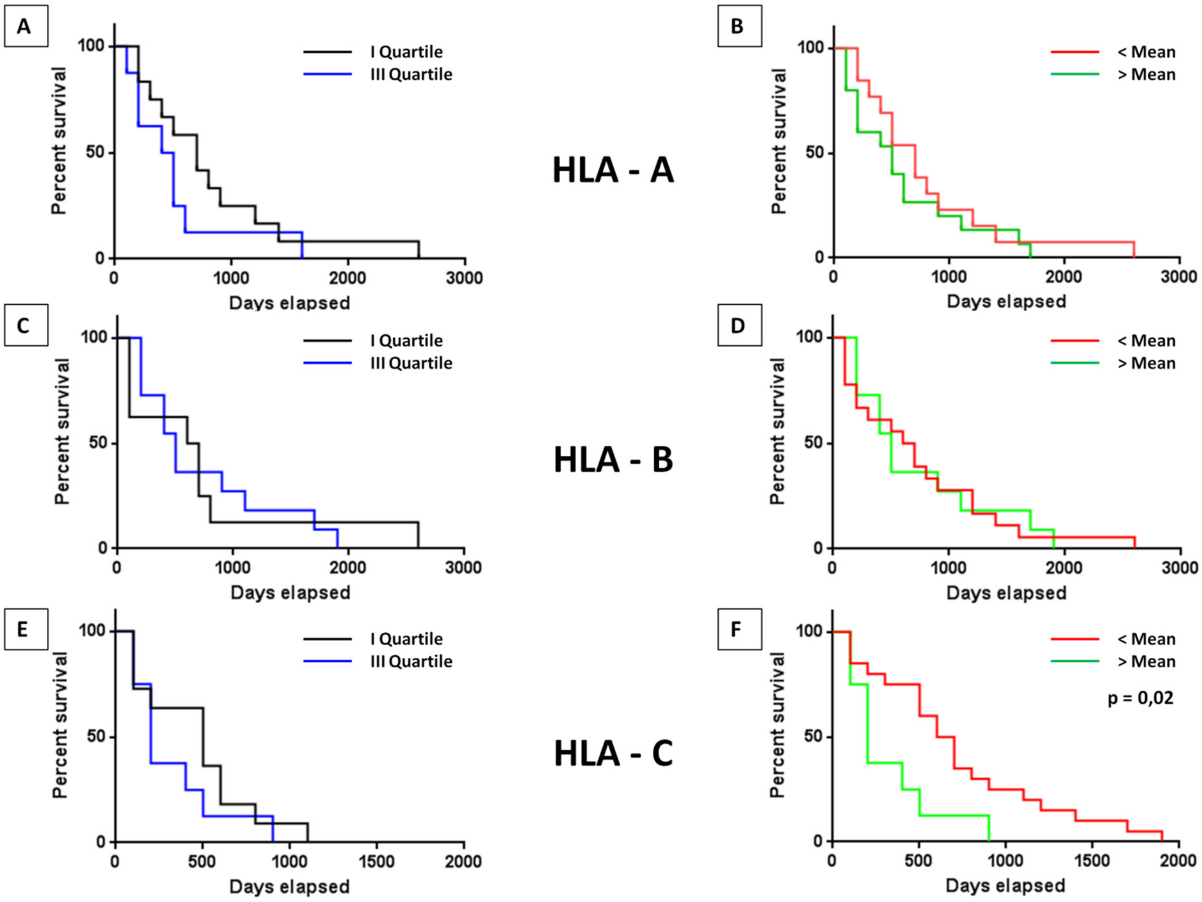

Figure S2. HLA-associated neoantigens and survival.

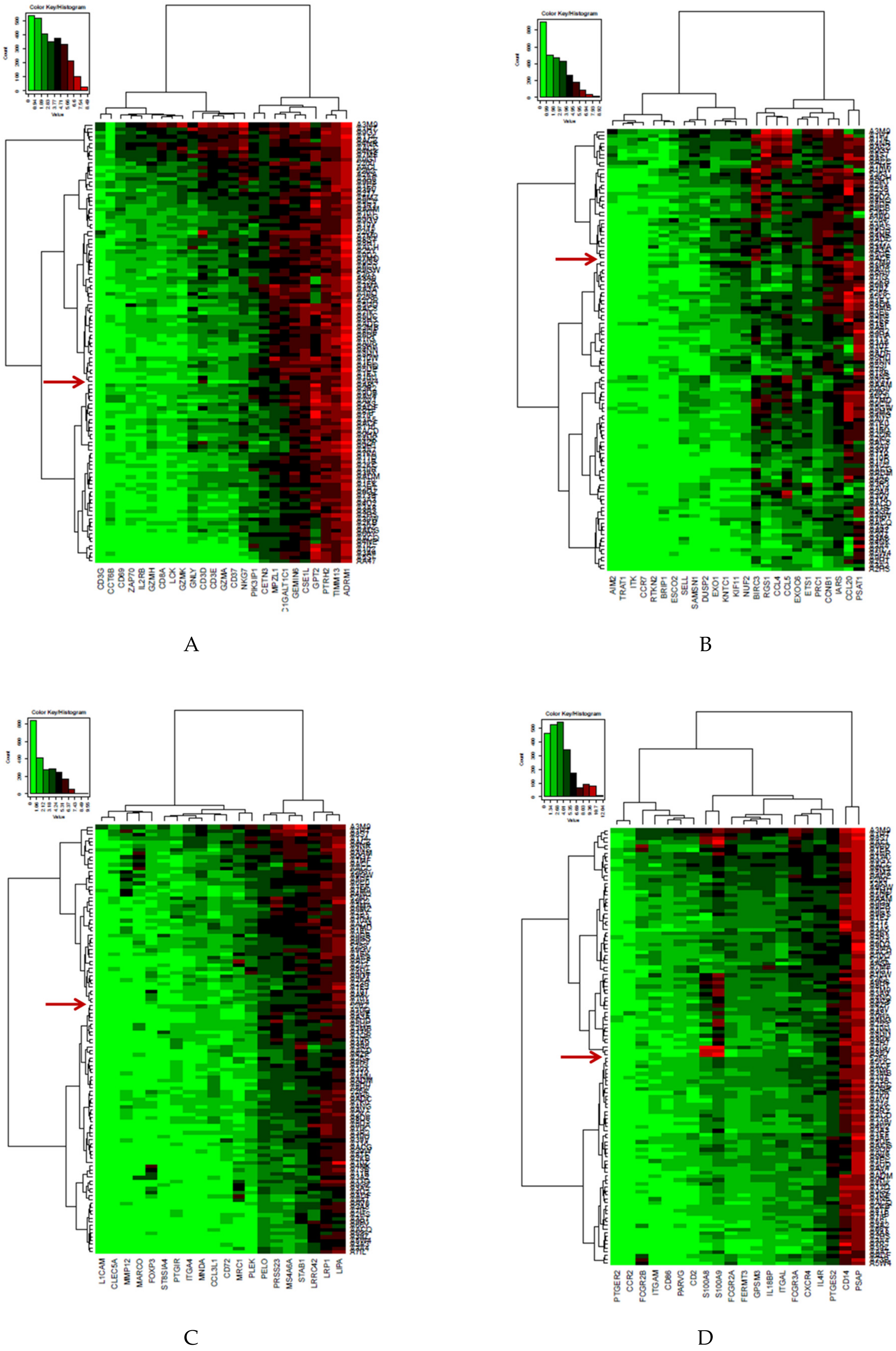

Figure S3. (**A**) Heat map of gene expression for tumor infiltrating CD8⁺ T cells. (**B**) Heat map of gene expression for tumor infiltrating CD4⁺ T cells. (**C**) Heat map of gene expression for tumor infiltrating Tregs. cells. (D) Heat map of gene expression for tumor infiltrating MDSC.

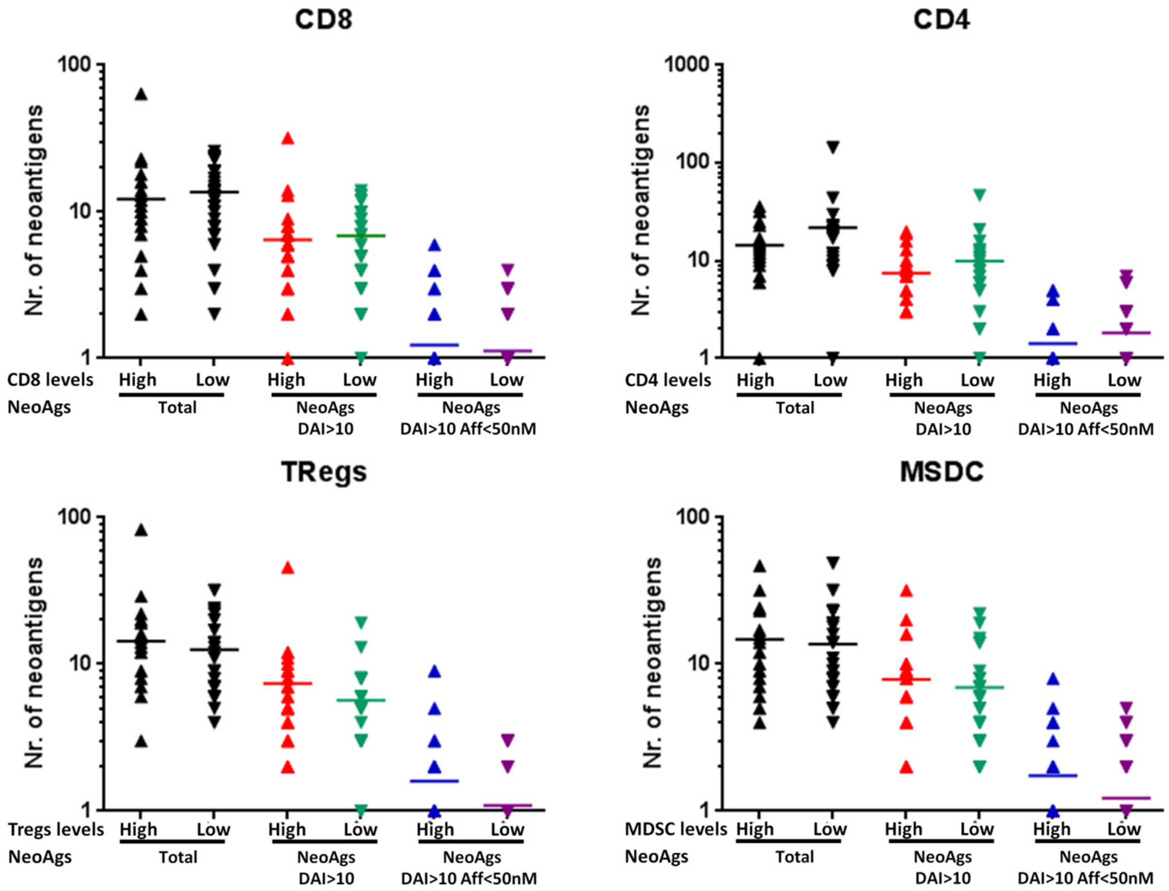

Figure S4. Correlation between neoantigens and tumor infiltrating cells.

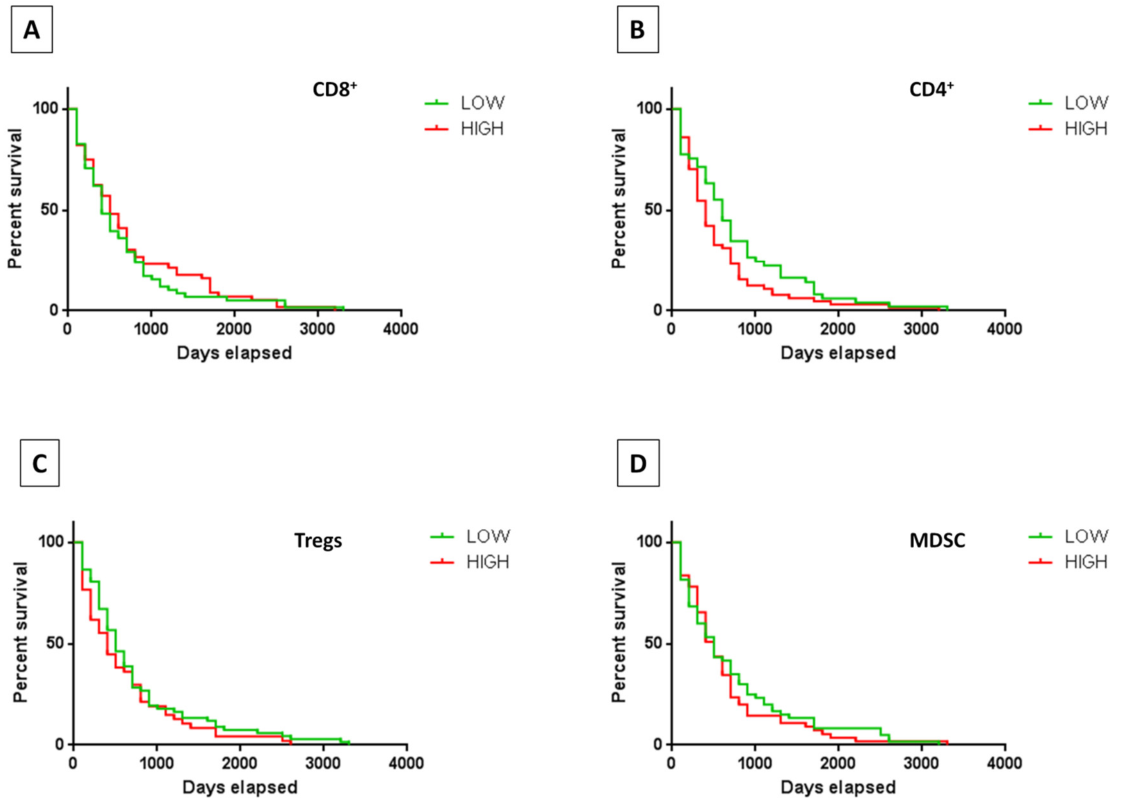

Figure S5. Tumor infiltrating cells and survival.

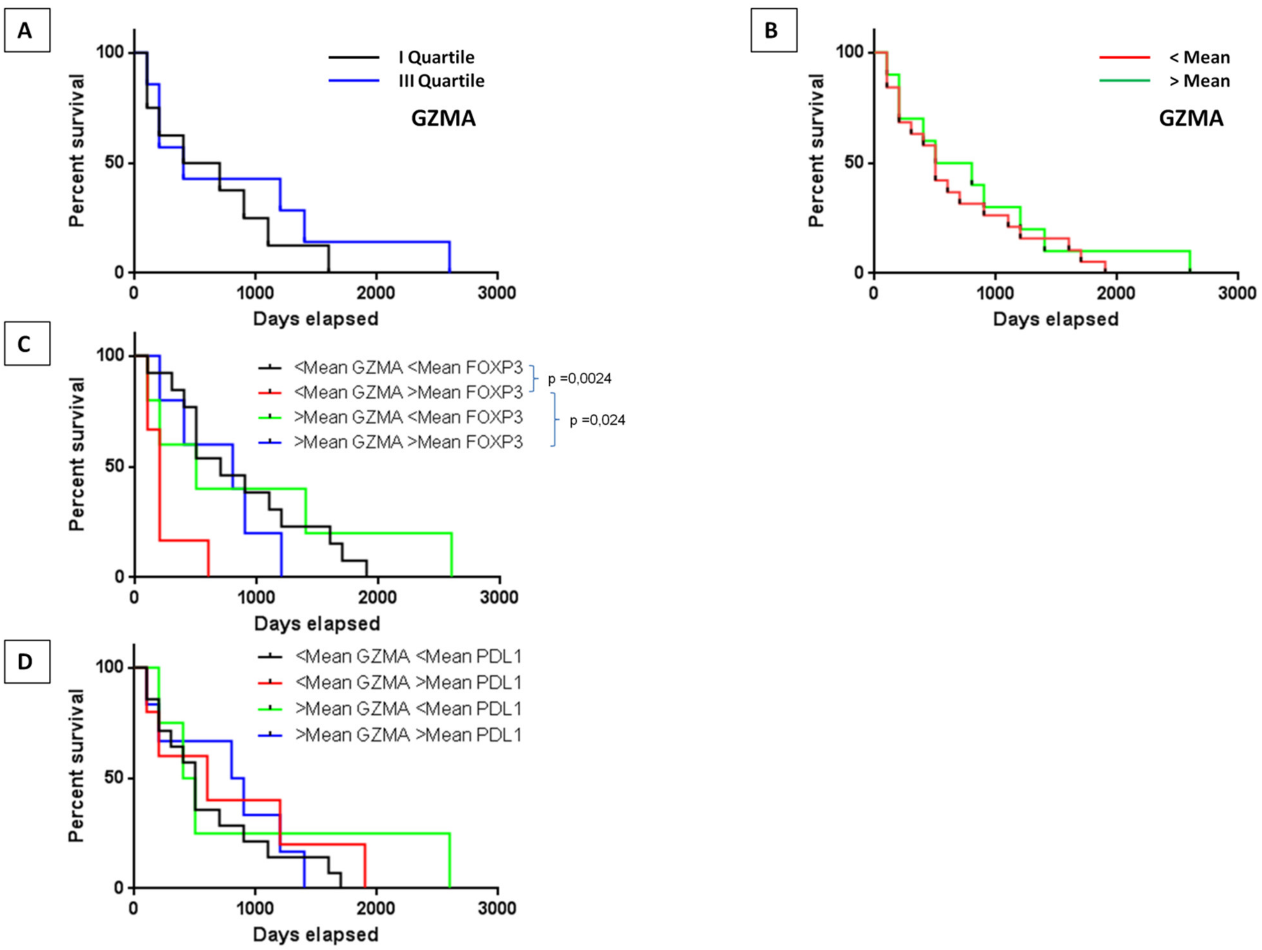

Figure S6. Tumor microenvironment and survival.

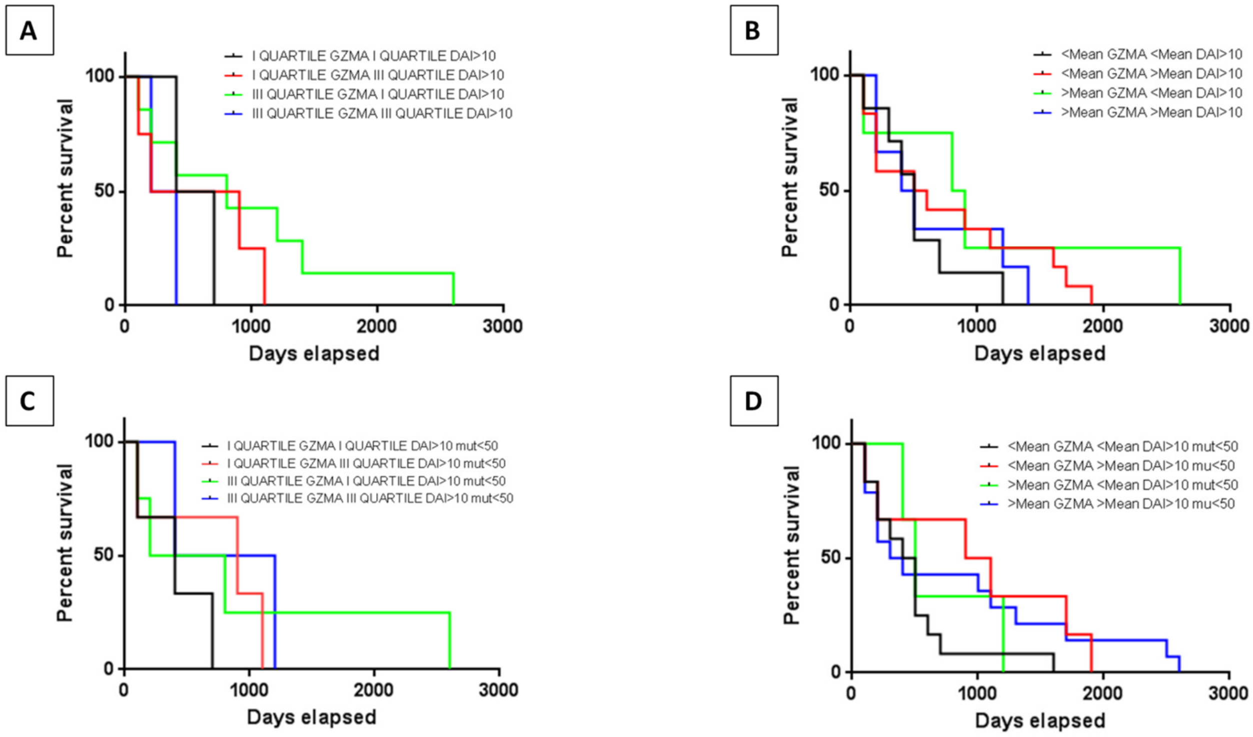

Figure S7. GZMA, neoantigens and survival.

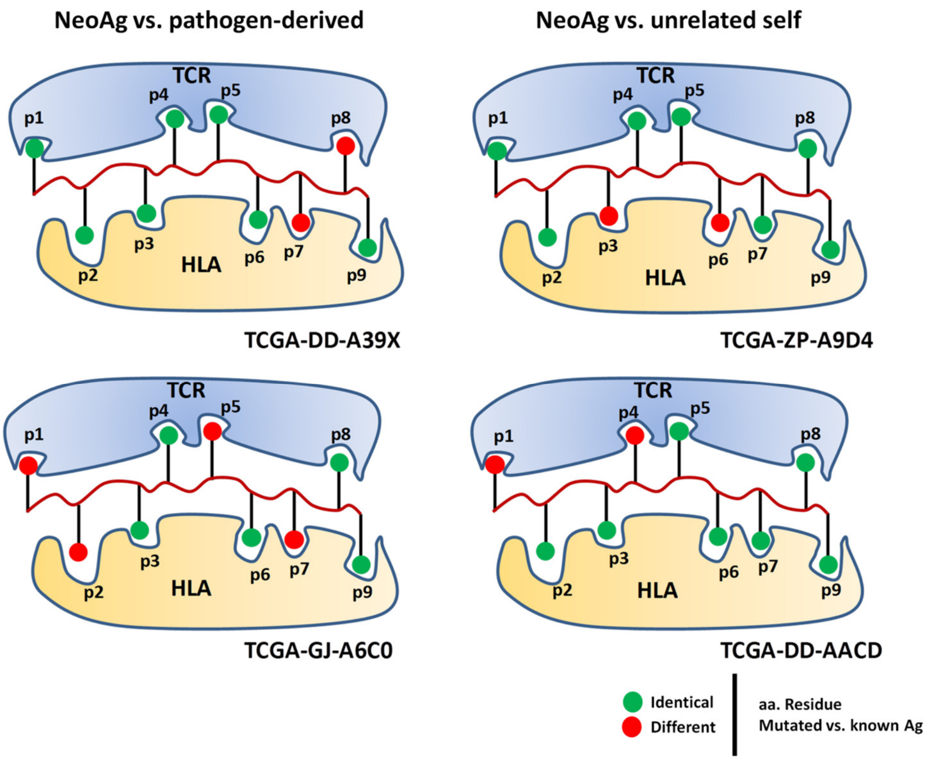

Figure S8. Sequence homology between neoantigens and published epitopes.
